# Comparative study of the neural differentiation capacity of mesenchymal stromal cells from different tissue sources: An approach for their use in neural regeneration therapies

**DOI:** 10.1371/journal.pone.0213032

**Published:** 2019-03-11

**Authors:** Daniela N. Urrutia, Pablo Caviedes, Rodrigo Mardones, José J. Minguell, Ana Maria Vega-Letter, Claudio M. Jofre

**Affiliations:** 1 Regenerative Cell Therapy Center, Clinica Las Condes, Santiago, Chile; 2 Program of Molecular & Clinical Pharmacology, ICBM, Faculty of Medicine, Universidad de Chile, Santiago, Chile; 3 Centro de Biotecnología y Bioingeniería (CeBiB), Departamento de Ingeniería Química, Biotecnología y Materiales, Facultad de Ciencias Físicas y Matemáticas, Universidad de Chile, Santiago, Chile; 4 Orthopedic Department, Clinica Las Condes, Santiago, Chile; 5 Program of Traslational Immunology ICIM, Faculty of Medicine, Clinica Alemana Universidad del Desarrollo, Santiago, Chile; Università degli Studi della Campania, ITALY

## Abstract

Mesenchymal stem cells (MSCs) can trans/differentiate to neural precursors and/or mature neurons and promote neuroprotection and neurogenesis. The above could greatly benefit neurodegenerative disorders as well as in the treatment of post-traumatic and hereditary diseases of the central nervous system (CNS). In order to attain an ideal source of adult MSCs for the treatment of CNS diseases, adipose tissue, bone marrow, skin and umbilical cord derived MSCs were isolated and studied to explore differences with regard to neural differentiation capacity. In this study, we demonstrated that MSCs from several tissues can differentiate into neuron-like cells and differentially express progenitors and mature neural markers. Adipose tissue MSCs exhibited significantly higher expression of neural markers and had a faster proliferation rate. Our results suggest that adipose tissue MSCs are the best candidates for the use in neurological diseases.

## Introduction

Mesenchymal stem cells (MSCs) are a class of adult stem cells, which undergo self-renewal and exhibit pluripotency [1]. In addition, MSCs have immunomodulatory properties, produce trophic factors for tissue repair/regeneration [2, 3], and differentiate into various cell lineages, including neurons and glial cells [4, 5].

MSCs were originally identified in the bone marrow [6], they have also been found in other locations such umbilical cord tissue [7], umbilical cord blood [8] adipose tissue [9] skin [10] teeth [11, 12] and pancreas [13]. Among all these tissues, adipose, skin and umbilical cord are attractive choices to obtain cells due to the relatively easy access to samples in clinical settings [10, 14–16].

Accordingly, MSCs properties have laid a solid foundation for their clinical application in the field of regenerative medicine [17, 18]. Furthermore, a precise characterization of MSCs derived from different tissues sources represent an essential requirement for the development of MSC-based therapies to repair and/or regenerate damaged tissues.

In the specific case of the central nervous system, nervous tissues show the most limited regeneration and recovery capabilities after injury. In humans, neurogenesis is restricted to the dentate gyrus of the hippocampus and, despite the existence of endogenous neural stem cells, their capacity is not enough to induce full repair and regeneration [19]. These facts account for the devastating nature of many neurological diseases where recovery is incomplete and major disability often results. Accordingly, the search for new sources of stem cells with potential to differentiate into a neural phenotype represents a central issue for the treatment of neurodegenerative conditions, post-traumatic and/or hereditary diseases.

In this regard, the promising results of animal and human studies using MSCs from several tissue sources [20–28], have presented the possibility of using these cells for neural repair. Nevertheless, *in vitro* studies using MSCs isolated from bone marrow and adipose tissue have shown variability in their ability to differentiate toward a particular mature neural lineage [29, 30], to generate functional neurons [31], as well as to support neural regeneration after transplantation [32]. Since these variations may result in heterogeneous clinical outcomes, there is a need to establish a relevant MSC source for neurological repair and regeneration.

In light of the above, the aim of this study was to evaluate the neural differentiation capacity of *ex vivo* expanded MSCs isolated from several human tissues, including adipose, bone marrow, skin and umbilical cord. The data from the studies described herein may be valuable for selecting the proper tissue source of MSC to be used therapeutically in neural regenerative therapies.

## Materials and methods

### 1. Collection and isolation of MSCs from the different tissue sources

This study was performed at the Regenerative Cell Therapy Center, Clinica Las Condes, Santiago, Chile. Procedures carried out in this study complied with regulations and were approved by the Research and Ethics Committees of Clinica Las Condes. All donors and/or their parents gave written informed consent for the use of the requested tissue. Average age of donors was 28 ± 5 years (with the obvious exception of umbilical cord), gender ratio (male/female) was 7:2 (Table 1). No donors used concomitant drugs.

**Table 1:** Tissue sources characteristics of MSCs utilized in this study.

Tissue Source	Donor patient age average	Donor patient gender n = 3	Subculture passage(#) used
Bone Marrow	27	All male	3–5
Adipose	28	2 female 1 male	3–5
Skin	29	All male	3–4
Umbilical Cord	new born	-	3–5

Tissue sources characteristics of MSCs utilized in this study. Donor patient age average and gender were n = 3.

Mesenchymal Stem Cells (MSCs) were obtained from Adipose Tissue (AT), Bone Marrow (BM), Skin, and Umbilical Cord (UC) (Table 1). For isolation of MSCs, the respective tissues were processed according to indications [33] for Bone Marrow-derived MSC (BM-MSC) and Adipose Tissue-derived MSC (AT-MSC), described in [34] for Umbilical Cord-derived MSC (UC-MSC), and indicated in [35] for Skin-derived MSC (SD-MSC). BM aspirates (n = 3) were obtained from the iliac crest. AT samples were obtained during abdominal plastic surgeries (n = 3). Ten-centimeter-long UC were collected and donated from consenting patients delivering full-term infants by caesarian section (n = 3). Pieces of skin tissue from arms (n = 3) were carefully dissected free of other tissue and cut into 2–3 mm^3^ pieces.

### 2. Culture and *ex vivo* expansion of MSCs obtained from the different tissues sources

MSCs-derived from the above-indicated tissue sources were cultured under the same culture conditions: growth medium, consisting in Minimum Essential Medium alpha modification (α - MEM, Gibco-Invitrogen, USA) supplemented with 10% Fetal Bovine Serum (FBS, Corning Cell Gro), 1X penicillin–streptomycin (Pen-strep, Biological Industries). As soon as a culture reached confluence, cells were expanded. In all studies, resulting MSCs at passage 3 to 5 were used.

### 3. Immunophenotyping

Cultures of Isolated MSCs obtained from the different origins were labeled with the following anti-human antibodies: CD11b-AF488, CD29-PE, CD73-PE, CD90-FITC, CD105-PE (BD Bioscience), CD34-PE, CD19-PE, CD45-FITC (Beckman C), and HLADR-PE from Invitrogen. Mouse isotype antibodies served as respective controls (Invitrogen). Labelled cells were analyzed using a FACS-Vantage-SE flow cytometry system running CellQuest software (BD). The fluorescence signals were collected using logarithmic amplification.

### 4. Population doubling time

To examine MSCs Population Doubling Time (PDT), cells at passage 3 were seeded at a density of 5X10^3^ cm^2^ and PDT calculated by using an algorithm available online (http://doubling-time.com) [36].

### 5. Adipogenic, chondrogenic and osteogenic differentiation

To assess adipogenic, chondrogenic and osteogenic differentiation, cells were cultured in basal medium until 70–80% confluence and then changed to every induction medium and stained [35, 37]. Images were obtained with microscope NIKON ECLIPSE Ti-s.

### 6. Neural induction

The induction protocol was adapted from [38–40]. Briefly, MSCs neural specification (step 1) was induced by culturing cells in α MEM supplemented with: 0,25X B27, 1X N2, 20 ng/mL EGF and 20 ng/mL FGF basic for 5 days. At the end of the neural specification treatment, MSCs were washed with PBS, and then neuronal commitment (step 2) was induced by exposing the cells to α MEM supplemented with 0,25X B27, 100 ng/mL Sonic HedgeHog, 2,5 μM Retinoic Acid and 1 mM AMPc during the next 10 days. Finally, we induced neuronal differentiation (step 3) adding 30 ng/mL BDNF during the final 3 days. One non-induced culture dish was also analyzed in every experiment as negative control. Neural Stem Cells (NSC) from StemPro were used as positive control and differentiated as indicated by the supplier. The cells were monitored continually after neuronal induction. The area in pixels were measured by ImageJ with “measure” function, and neurite quantification with NeuronJ pluggin.

### 7. Reverse transcription polymerase chain reaction RT–PCR

To detect gene expression indicative of MSCs neural differentiation, mRNA was harvested using trizol (Life Technologies). cDNA was synthesized from the extracted mRNA using the Verso cDNA Kit (Thermo Scientific). RT-PCR analysis was then performed with Brilliant III SYBR GREEN Q-PCR (Agilent), with primers (S1 Table) for NESTIN neurofilaments NEFM and NEFL, NURR1, S100B, SAP90 and NT3. The housekeeping gene used was GAPDH. The following amplification parameters were utilized for the q RT-PCR: 10 minutes at 95°C, 40 cycles of 30 s at 95°C, 30 s at 55°C and 30 s at 72°C, followed by one cycle of 10 s at 95°C, 5 s at 25°C, 1 s at 70°C and 1 s at 95°C. Results were analyzed using the 2^-ΔCt^ method relative gene expression to GAPDH.

### 8. Immunocytochemistry

MSCs were assayed as described preciously [41]. Nuclei were counterstained with DAPI. The primary and secondary antibodies used are shown in S2 Table. The conditions were maintained in negative controls. The dishes were examined under a fluorescence microscope (NIKON ECLIPSE Ti-s). ImageJ software (National Institute of Health) was used to pseudo-color images, adjust contrast, and add scale bars.

### 9. Synaptic vesicle accumulations

To visualize synaptic vesicle accumulations, after 18 days of neural induction, MSCs from all sources were loaded with 4 μM styryl dye SynaptoRed C2 {4-[6-[4-(Diethylamino) phenyl]-1,3,5-hexatrien-1-yl]-1-[3-(triethylammonio) propyl] pyridinium dibromide, FM 4–64 molecular probes, Tocris Bioscience} in depolarizing extracellular solution (80 μM) during 120 seconds. After loading, cells were washed witch HBSS 1X during 5 min. Cells were imaged immediately under fluorescence microscope NIKON ECLIPSE Ti-s.

### 10. Statistical analysis

All results are based on at least three independent experiments and are expressed as mean ± SEM or SD for three MSCs donors in each group. Statistical significance for PDT and CTCF analysis was determined using t-student test. In RT-PCR analysis of relative expression and area and neurite outgrowth measurements one-way ANOVA was used, followed by a Bonferroni multiple comparison test using Prism5 software (GraphPad, La Jolla, CA).

## Results

### 1. Characterization of MSCs Isolated from different tissues

#### a) Morphology

Once in culture, MSCs from all sources were relatively homogeneous in morphology with a characteristic fibroblastic-like morphology when attached to culture plastic dishes (S1 Fig)

#### b) Immunophenotype

MSCs were tested for analysis of expression of different markers by flow cytometry. Results (S2 Fig) indicate that cells from all tissue sources were negative for hematopoietic marker (CD34), leucocitic markers (CD11b, CD19 and CD45), and HLA-DR (Human Leukocyte Antigen). In turn, were positive for specific MSCs markers (CD73, CD105) and cell adhesion markers (CD29, CD90). In all different donors and tissue origin, the above phenotype was consistent, thus confirming the MSCs phenotype previously described [42–45].

#### c) Adipogenic, chondrogenic and osteogenic differentiation

MSCs isolated from AT, BM, Skin and UC were capable to differentiate into adipogenic (S3 Fig), chondrogenic (S4 Fig) and osteogenic (S5 Fig) lineages which is consistent with the minimal characterization criteria of MSCs [42].

#### d) Proliferation

**Fig 1:**
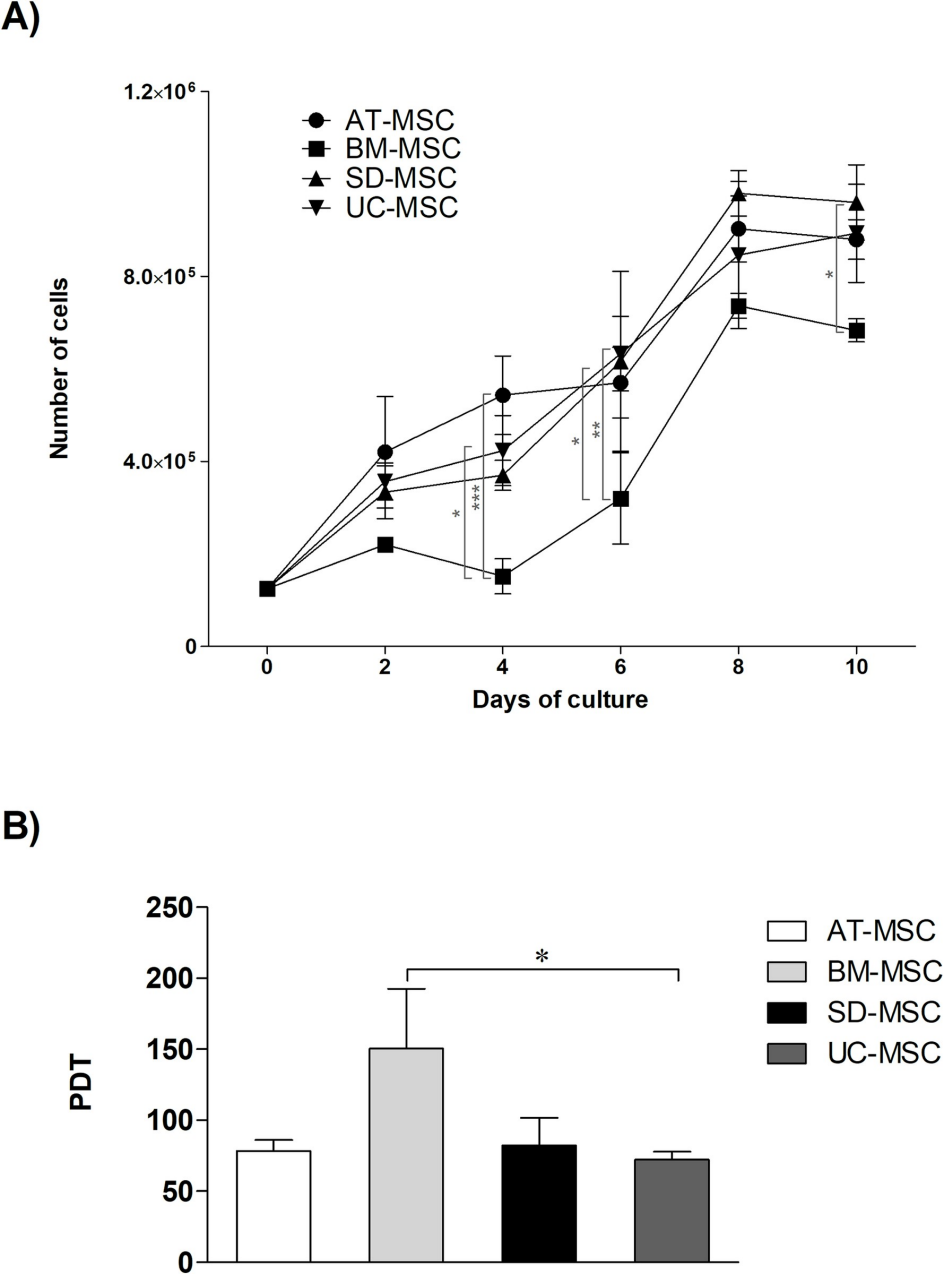
Proliferation of MSCs from different sources. Proliferation capability of AT-MSC, BM-MSC, SD-MSC and UC-MSC. **A)** Growth curve and **B)** Population doubling time. ns P> 0.05, * P <0.05, ** P <0.01, *** P <0.001.

As shown in the growth curve in Fig 1A, MSCs from all sources, after undergoing an adaptation period to culture conditions during the first 4 days, enter to a logarithmic phase, and later on day 8, exhibit contact inhibition and reach the plateau phase. The Population Doubling Time (PDT) calculated at day 6 (Fig 1B) showed that UC-MSC had reaches a larger population in less time (±72.77 hours), which means that has greater proliferation capacity, followed by AT-MSC (±78.37 hours) and SD-MSC (±82.11 hours) which did not show significant differences. Moreover, BM-MSC show the lowest PDT (±150.52 hours) which was statistically significant.

### 2. Assessment of mesenchymal stem cell neurogenic potential

Human MSCs previously characterized from the all sources were subjected to neuronal induction medium during 18 days. To investigate whether AT-MSC, BM-MSC, SD-MSC and UC-MSC exhibited neurogenic differentiation capabilities, we compared morphology changes and expression of neural markers at mRNA and protein levels during neural induction.

#### 2a) Morphologic change after neural induction

**Fig 2:**
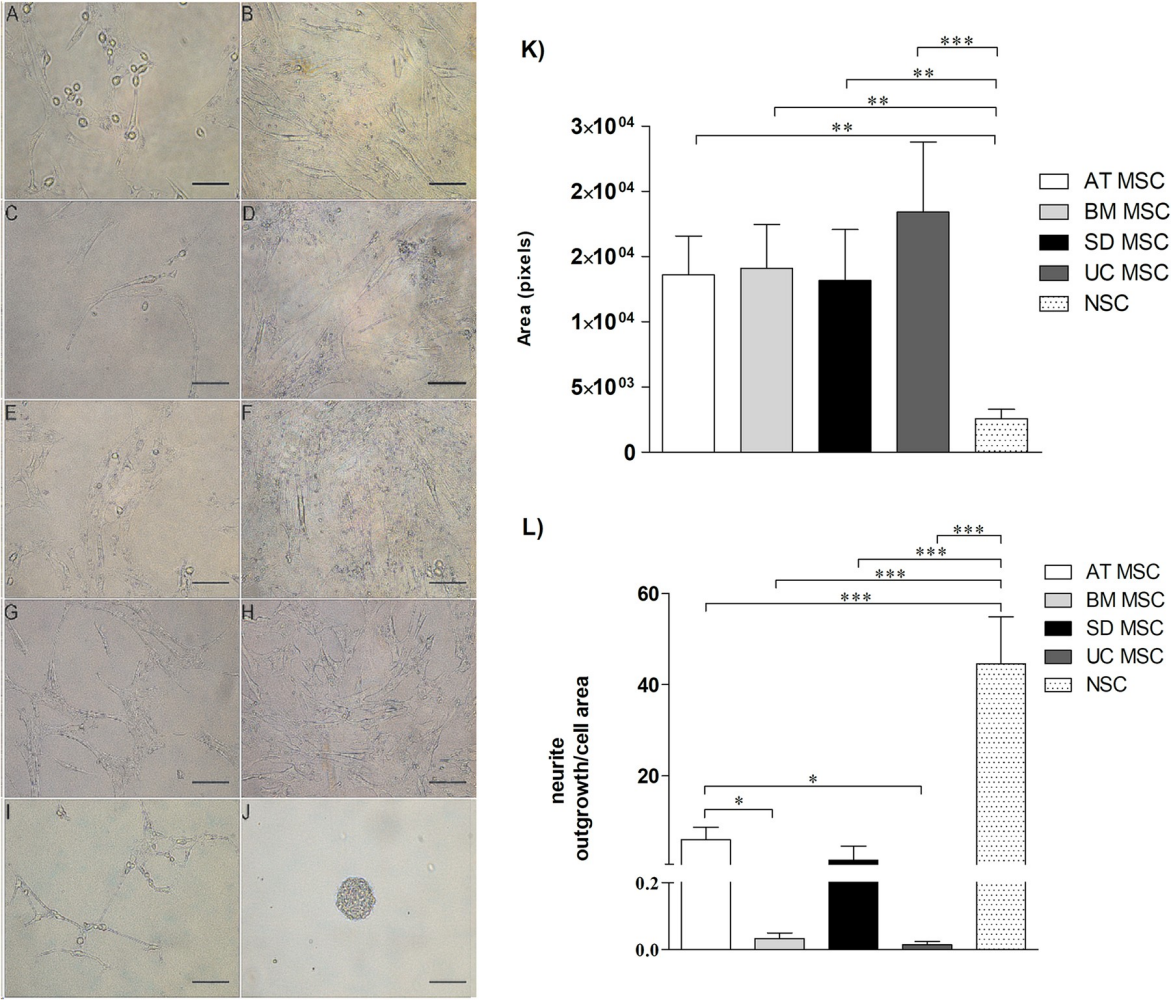
Changes in MSC morphology. Changes in MSC morphology after 18 days of neural differentiation: **(A, B)** AT-MSC, **(C, D)** BM-MSC, **(E, F)** SD-MSC, **(G, H)** UC-MSC, **(I, J)** Positive control (NSC). Scale bar 100 μm. Induced MSC (**A, C, E, G**) adopt neural-like morphology as well as cytoplasm retraction towards the nucleus, which is more notorious in A y E compared to negative controls (**B, D, F, H)** and were quantified in **(K)** Area (pixels) and **(L)** Number of cells with visible neurite outgrowth. Data represents means ± SEM of 3 separate experiments. ns P> 0.05, * P <0.05, ** P <0.01, *** P <0.001.

To assess MSCs neurogenical potential, we analyzed the morphological change in neural induced MSCs. Following 18 days in neural induction medium [39], MSCs from different sources (AT, BM, Skin, UC) changed their morphology from flat, spindle-shaped cells to neural-like cells which included retraction of the cytoplasm towards the nucleus and several cytoplasmic extentions, similar to those exhibited by positive control neural stem cells (Fig 2A–2E). This change is more distinguishable in SD-MSC and AT-MSC (Fig 2A and 2E), which also reduced their sized, confirmed by the quantification of area (Fig 2K) and number of visible neurite outgrowth (Fig 2L).

#### 2b) Expression of neural markers

**Fig 3:**
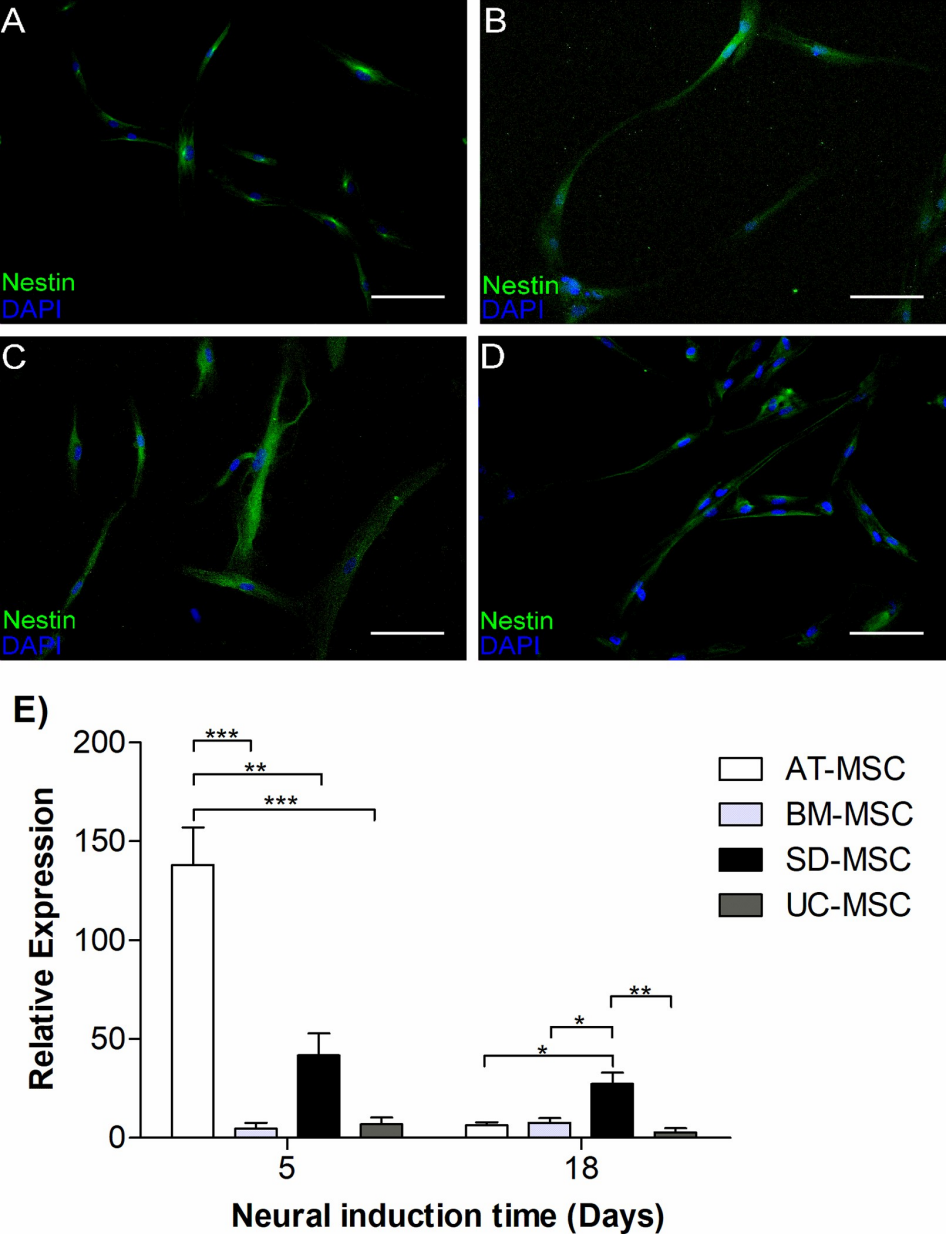
Nestin expression in induced MSC. Nestin relative expression in AT-MSC, BM-MSC, SD-MSC and UC-MSC after neural induction. **(A, B, C, D)** Immunocytochemistry analysis of nestin at day 5 of neural induction; **(A)** AT-MSC, **(B)** BM-MSC, **(C)** SD-MSC, **(D)** UC-MSC. Nestin marker expression (green) and nuclei (Blue), scale bar 100 μm. **E)** Time dependent expression of *nestin* assessed by RT-PCR. Data is presented as an average of three independent patient samples and error bars represent mean ± SEM, ns P> 0.05, * P <0.05, ** P <0.01, *** P <0.001.

Our results of immunofluoresence analysis confirmed the expression of nestin at day 5 of neural induction (Fig 3A–3D). Since nestin is a progenitor marker, its expression was analyzed at early stages of neural induction (day 5) and compared with at a later stage (day 18, Fig 3E) by RT-PCR. Our results showed that nestin expression at day 5 was higher as compared with expression at day 18 (Fig 3E). Further, nestin expression was considerably higher in AT-MSC as compared to BM-MSC, SD-MSC and UC-MSC.

**Fig 4:**
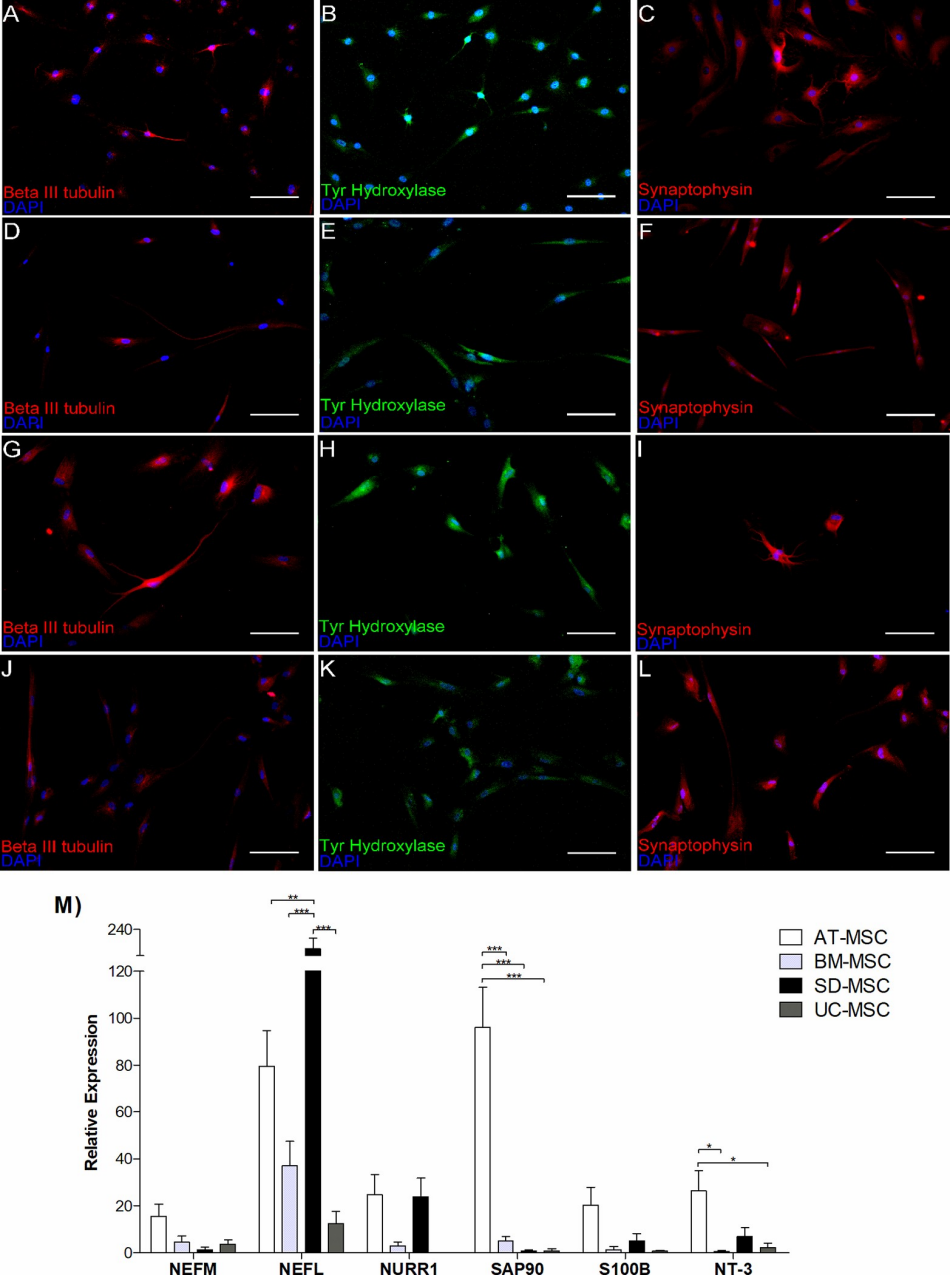
Expression of neural lineage related markers after neural induction. Relative expression of related markers after neural induction in MSC from different sources. **(A-L)** Immunocytochemistry analysis of induced MSC: **(A-C)** AT-MSC, **(D-F)** BM-MSC, **(G-I)** S-MSC, **(J-L)** UC-MSC, showing protein expression of neural specific markers: βIII tubulin (red), tyrosine hydroxylase (green) and synaptophysin (red). Scale bar 100 μm. **M**) Comparative analysis of mRNA expression levels of neural markers, NEFM, NEFL, Nurr1, Sap90, S100b and NT-3. Relative gene expression of each gene were normalized to the expression of the housekeeping gene GAPDH. Data represents means ± SEM of 3 separate experiments. ns P> 0.05, * P <0.05, ** P <0.01, *** P <0.001.

MSC from all sources express βIII tubulin, as measured by immunocytochemistry (Fig 4A–4L), as well as the dopaminergic marker tyrosine hidroxilase, estructural marker βIII tubulin and synaptic marker synapthophysin, at 18 day of neural induction. At day 18 of neural induction the expression of neurofilament genes *NEFL* and *NEFM* was higher in AT-MSC and SD-MSC, respectively, compared to that of BM-MSC and UC-MSC. Moreover, AT-MSC showed highly superior gene expression of the dopaminergic neuron marker NURR1, astrocyte marker S100B and neurotrophic factor NT-3, which was statistically significant (Fig 4M).

**Fig 5:**
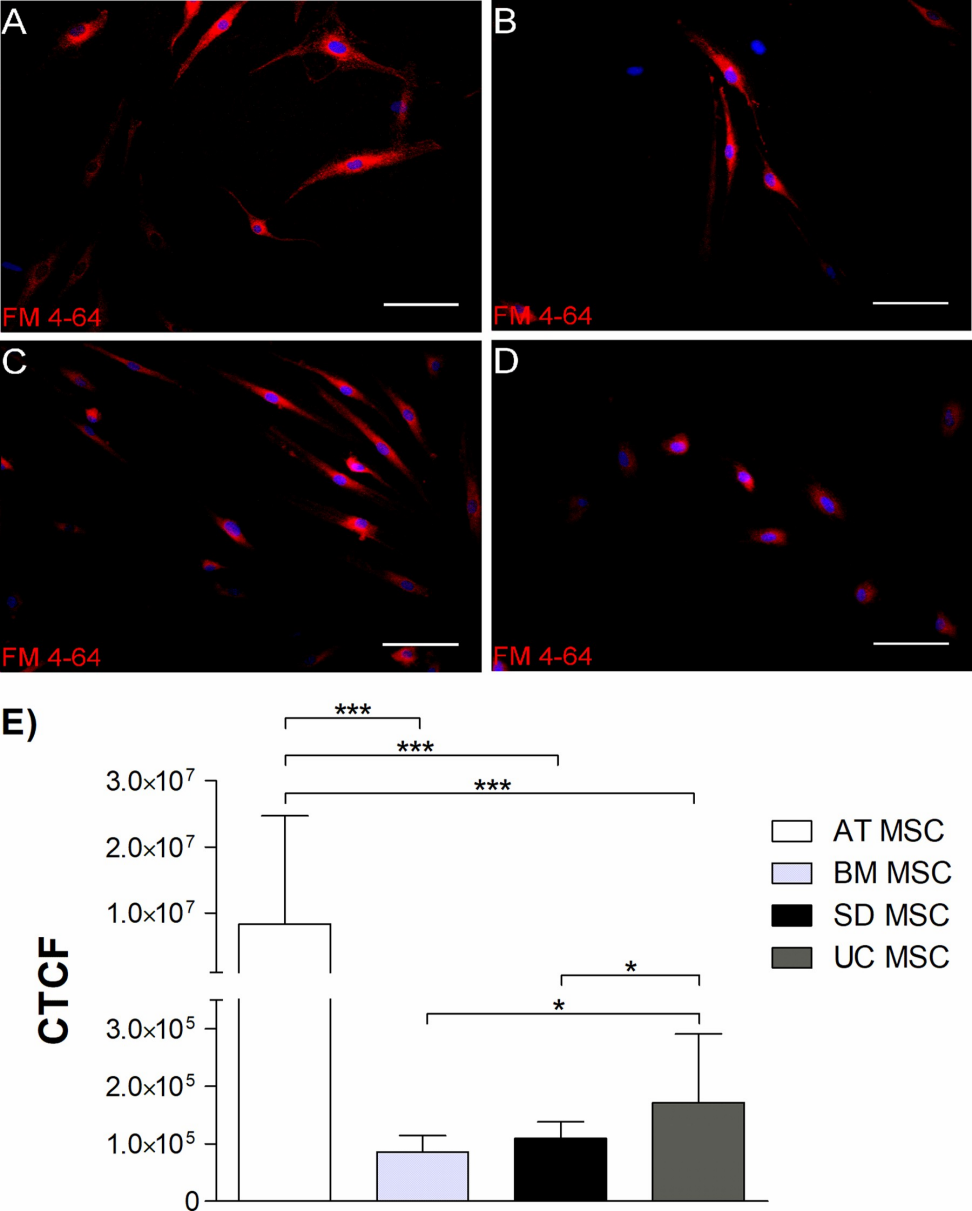
Synaptic vesicle staining after 18 days of neural differentiation. FM 4–64 dye was positive in neural-induced MSC. (A) AT-MSC, (B) BM-MSC, (C) SD-MSC, (D) UC-MSC. Nestin marker expression (green) and nuclei (Blue), Scale bar 100 μm. (E) Corrected Total Cell Fluorescence (CTCF), Data represents means ± SEM of 3 separate experiments. ns P> 0.05, * P <0.05, ** P <0.01, *** P <0.001.

We additionally detected the labeling of FM 4–64 (synaptored C2) that becomes fluorescent when incorporated into plasma membrane, used to follow up synaptic activity in induced MSC from all studied sources (Fig 5) and quantified, showing that AT-MSC has higher fluorescence intensity against BM, Skin and UC (Fig 5E).

## Discussion

The objective of this study was to provide a comparison of the capability of MSCs isolated from several human tissues, to differentiate under *in vitro* conditions to neuron-like cells, evidenced by morphological changes and by the expression of neural markers.

It is well known that several donor characteristics such as age, gender, underlying medical conditions and/or use of concomintant drugs, affect the functional properties of MSCs [46–49]. Therefore, to minimize the putative effect of the above mentioned factors on MSCs neural differentiation capacity, in this study, human tissues were solely obtained from healthy donors of similar age (Table 1).

The results reported here suggest that MSCs isolated from adipose tissue, bone marrow, skin and umbilical cord tissue share common cell surface epitopes as well as an ability to undergo multilineage mesenchymal differentiation (S2–S5 Figs).

### Proliferative of MSCS isolated and *ex vivo* expanded from different tissue sources

The proliferative capacity of AT-MSC and UC-MSC (Fig 1), was higher than SD-MSC and BM-MSC. These differences suggest a cell culture heterogenicity, including a variable proportion of self renewing cells, versus lineage-commited cells in different tissue source stromal cell compartment [50,51]. These results could be significant in the election of a tissue source of MSCs, intended to be used in cell-based therapies, which need a viable and ample number of cells to be procured in less time in order to achieve a successful clinical outcome [52].

### Assessment of the capacity of MSCs from diverse sources to differentiato into neural-like cells

Results of neural induction of MSCs obtained from different human tissue-sources, indicated that after 18 days of exposure to a neural induction medium, MSCs morphology changed from a spindled to a neuron-like shape (Fig 2). Beyond these morphologycal changes, indicative of MSCs neural differentiation, we also studied the onset of a meaningful group of neural markers, including *nestin*, *nefm*, *nefl*, *sap90*, *nurr1*, *s100b*, *nt3*, βIII tubulin, tyrosine hydroxylase and synaptophisin (Figs 3 and 4).

The neural induction medium applied during differentiation comprises the use of substances that induce the molecular expression pattern that occurs during adult neurogenesis of neur al stem cells, where neuronal differentiation and maturation occur in three steps: -neural undifferentiated progenitors, -immature neurons expressing early neuronal genes and finally, mature neurons expressing late neuronal genes [53, 54].

To asses this process, we first analyzed nestin expression by MSC, neural stem cell marker that can be considered as a primary evidence of the capability of these cells to generate neural progenitors [55]. The above in turn is sustained by the observed time-dependent expression of *nestin* at day 5, which descreased after 18 days of induction (Fig 3A and 3B).

To evaluate whether MSCs also express a potential to generate immature neurons, we confirmed the expression of βIII tubulin (Fig 4A, 4D, 4G and 4J), an indicator of neural commitmnent [56]. Finally, when cells receive BDNF neurogenic stimulation, they differentiated and acquired the mature phenotype expressing NEFL and NEFM (Fig 4M) which are asossiated with neural maturation [57, 58].

### Assesstment of synapse, astrocytes, dopaminergic and neurotrophic markers

In order to demonstrate the protein expression related to synapse formation, we investigated whether AT-MSC, BM-MSC, SD-MSC and UC-MSC were capable of expressing the pre-synaptic protein synaptophysin (Fig 4C, 4F, 4I and 4L), and the post-synaptic protein SAP 90 (PSD95) (Fig 4M). We demostrated that MSCs from all different sources express synaptophysin as evidence of synapsis [59, 60], however AT-MSC showed the highest expression of *sap90*, suggesting that the latter cell type would be the most likely to form a synaptic structure. This results are confirmed in Fig 5A–5E, where AT-MSC show the highest CTCF (Corrected total cell fluorescence) which could probably indicate the presence of functional presynaptic terminals [61].

Results related to the expression of *s100b*, an astrocyte marker, showed that MSCs are capable of differentiating not only into neurons but also into astrocytes. As previously described, adult NSC are specialized astrocytes in others parts of the brain [62]. Astrocytes has the potential to promote neurogenesis in the adult hippocampus [63]. All this considered, and in agreement with our results, we suggest that AT-MSC has higher potential to form astrocytes (Fig 4M).

Moreover, we explored if MSCs were capable to express dopaminergic neuron markers *in vitro*, to considere it as a supplying cell source for the treatment of neurodegenerative conditions like Parkinson disease, in that regard, expression of *nurr1* and tyrosine hydroxylase were analyzed. Accordingly, MSCs from all different sources were capable of expressing both markers, suggesting a committed neuronal phenotype [64].

Additionally, we evaluate neurotrophic potential quantifying the expression of *neurotrophin 3* (*NT-3*) at mRNA level. The above is important since growth-factors-mediate the activation and/or mobilization of endogenous stem cells as well as the reparative action of MSCs [24, 65]. Results show that AT-MSC had the highest expression of NT-3, suggesting that this cell type could exert a better neurotrophic effect *in vitro* (Fig 4M), however, measurement of neurotrophic secretion is still need it to define the role of NT3 in differentiation and trophic effects of neural-induced MSCs.

The above differences in neural marker expression between induced-MSCs could be explained by observing aspects that might affect the neural differentiation potential; these include signalling pathways and transcription factors involved in neuronal fate: the same pathways involved in NSC-neurogenesis are involved in the regulation of MSC chondrogenesis or osteoblastic differentiation [66–68] which could generate different population of differentiated cells. Besides, it has been demostrated that naïve MSCs already express neural-linked markers [69–70], which could indicate the presence of an heterogeneous population of cells and it is possible that the neural differentiation of MSC *in vitro* are due to the contribution of some neural-committed progenitors already present in the culture rather than the neural differentiation of the whole population [71]. Additionally we have to consider the evidence that showing the expression of proteins typical of nervous tissue in stromal cells, such as cathecolamines [72] neurotrophic factor receptors [73] and/or synaptic proteins [74] which could also generate a different neural marker expression pattern.

In summary, the results of this study indicate that after *in vitro* neural induction, MSCs from all analized tissue sources, slightly differ in morphology, phenotipic characteristics and in their potential to differentiate into neuron-like cells. However, AT-MSC proliferate significantly faster, generated neuron-like cells expressing higher levels of neural markers (Figs 1 to 5). Moreover, previous studies [31, 75] have shown that AT-MSC exhibited an eletrophysiologycal response after neural induction, characteristic of mature-functional neurons and fundamental for signal transmission in the nervous system. Additionaly, adipose tissue is one of the most advantageous sources of MSCs, due to their accessibility and easy of isolation. Finally, AT- MSCs have biological advantages in their proliferative capacity, pattern of secreted proteins (basic fibroblast growth factor, interferon-γ, and insulin-like growth factor-1) and immunomodulatory effects [76], showing as emerging and attractive option for stem cell using therapies.

## Conclusion

In the present report, neural regenerative therapy using MSCs obtained from different tisssue sources appears as a feasible and a promissing clinical option for the treatment of neurological affections. From the cell types tested, AT-MSC figure as the most appealing cell source, due to it ease of access and faster proliferation rates. Despite our results represent a novelty comparison between attractive sources of MSCs, there is a need for demostrate a genuine and complete neuronal differentiation, based on those criteria that define a neuronal cell [77] which is only probable by functional assays of synaptic transmission, membrane potential and functional action potential [78]. Also, it is evident that future studies are needed to further optimize and maximize the quality, efficacy and safety clinical use of MSCs [79].

## Supporting information

S1 Table Primers utilized in RT-PCR. Description of primers utilized in the RT PCR analysis, name of the gen, sequence, melting temperature, product size (bp) and database code.
(PDF)

S2 Table Antibodies utilized in immunocytochemistry. Description of the antibodies utilized in the study. Including name, provider and concentration used in the procedure.
(PDF)

S1 Fig Fibroblastic-like morphology and adherence to plastic of MSCs from different tissue sources. Fibroblastic-like morphology and adherence to plastic evidence of A) AT-MSC, B) BM–MSC, C) SD-MSC, D) UC-MSC. Scale bar 100 μm.
(PDF)

S2 Fig Immunophenotype of MSCs from different tissue sources. Histograms showing antigen expression in freshly (%): (A) AT-MSC, (B) BM-MSC, (C) SD-MSC and (D) UC-MSC. From left to right CD19, CD44, CD45, CD90, HLA-DR, CD29, CD73 CD105, CD73, CD34, CD105, CD11b. Black filled histogram: antigen expression; solid red line: auto-fluorescence control.
(PDF)

S3 Fig Adipogenic differentiation of MSCs from different tissue sources. Adipogenic differentiation of MSCs from A,B) AT-MSC, B,C) BM–MSC, C,D) SD-MSC, D,E) UC-MSC. Negative controls (B,C,D,E). All were stained with Oil Red O. Scale bar 100 μm.
(PDF)

S4 Fig Chondrogenic differentiation of MSCs from different tissue sources. Adipogenic differentiation of MSCs from A,B) AT-MSC, B,C) BM–MSC, C,D) SD-MSC, D,E) UC-MSC. Negative controls (B,C,D,E). All were stained with Safranin O. Scale bar 100 μm.
(PDF)

S5 Fig Osteogenic differentiation of MSCs from different tissue sources. Adipogenic differentiation of MSCs from A,B) AT-MSC, B,C) BM–MSC, C,D) SD-MSC, D,E) UC-MSC. Negative controls (B,C,D,E). All were stained with Alizarin Red. Scale bar 100 μm.
(PDF)
