# Supplementary material for: Comparative study of the neural differentiation capacity of mesenchymal stromal cells from different tissue sources: An approach for their use in neural regeneration therapies
Source: PLoS One. 2019 Mar 11;14(3):e0213032. doi: 10.1371/journal.pone.0213032 (PMC6437714; doi:10.1371/journal.pone.0213032)
Supplement: S1 Table — Description of primers utilized in the RT PCR analysis, name of the gen, sequence, melting temperature, product size (bp) and database code. (PDF) [file pone.0213032.s001.pdf]

Supporting information files

S1 Table: primers utilized in RT-PCR

Gen	Sequence *Forward* (F); *Reverse* (R)	Tm °C	Product (bp)	database
**Nestin**	F: CAACAGCGACGGAGGTCTC<br>R: GCCTCTACGCTCTCTTCTTTGA	62,4<br>61,5	164	NM006617
**NEFM** *(Neurofilament medium polypeptide)*	F: GAAATCGCTGCGTACAGAAAAC<br>R: TAATGGCTGTCAGGGCCTCTT	60,4<br>63,0	241	NM001105541
**NEFL** *(Neurofilament light polypeptide)*	F: CGACAGCTTGATGGACGAAAT<br>R: GATCTGCGCGTACTGGATCTG	60,7<br>62,6	91	NM006158.3
**Sap90** (*Synapse-Associated Protein 90)*	F: CACAACCTCTTATTCCCAGCAC<br>R: CATGGCTGTGGGGTAGTCG	60,9<br>62,1	79	NM001365
**Nurr1** (*Nuclear receptor related 1*)	F: GGGCTGTGTAAGCAGAACG<br>R: AAGGCCCGAATCTCAGGCT	60,7<br>63	107	NM199293
**S100b** (*S100 calcium-binding protein B)*	F:TGGCCCTCATCGACGTTTTC<br>R:ATGTTCAAAGAACTCGTGGCA	62.5<br>60.2	248	NM006272.2
**NT- 3** *(Neurotrophic factor)*	F: CGGATGCCATGGTTACTTTTG<br>R: CCTTGGATGCCACGGAGATA	54.5<br>56.7	100	NM_002527
**GAPDH** *(Glyceraldehyde-3-Phosphate*	F: AAGGTGAAGGTCGGAGTCAAC<br>R:GGGGTCATTGATGGCAACAATA	61,7<br>60,6	102	NM_002046

Dehydrogenase)				
