# Supplementary material for: Comparative study of the neural differentiation capacity of mesenchymal stromal cells from different tissue sources: An approach for their use in neural regeneration therapies
Source: PLoS One. 2019 Mar 11;14(3):e0213032. doi: 10.1371/journal.pone.0213032 (PMC6437714; doi:10.1371/journal.pone.0213032)
Supplement: S2 Table — Description of the antibodies utilized in the study. Including name, provider and concentration used in the procedure. (PDF) [file pone.0213032.s002.pdf]

Supporting information files

S2 Table: antibodies utilized in immunocytochemistry

Antibody/dye	Provider	Concentration used
DAPI	Thermo Fisher 62247	1:1000
Anti-human nestin	Millipore MAB5326	1:500
anti-human βIII tubulin	RyD Systems MAB1195	5 µg/mL
Anti-human tyrosine hydroxylase	Nobus Biologicals NB300-109	1:300
Anti-human Synaptophysin	RyD Systems AF5555	5 µg/mL
donkey anti rabbit NL 493	RyD Systems	1:200
donkey anti mouse NL493	RyD Systems	1:200
donkey anti goat Alexa 488	Abcam	1:200

Table S2: Description of the antibodies utilized in the study. Including name, provider and concentration used in the procedure.
