# Supplementary material for: Comparative study of the neural differentiation capacity of mesenchymal stromal cells from different tissue sources: An approach for their use in neural regeneration therapies
Source: PLoS One. 2019 Mar 11;14(3):e0213032. doi: 10.1371/journal.pone.0213032 (PMC6437714; doi:10.1371/journal.pone.0213032)
Supplement: S1 Fig — Fibroblastic-like morphology and adherence to plastic evidence of A) AT-MSC, B) BM–MSC, C) SD-MSC, D) UC-MSC. Scale bar 100 μm. (PDF) [file pone.0213032.s003.pdf]

Supporting information files

Figure S1: Fibroblastic-like morphology and adherence to plastic of MSCs from different tissue sources.

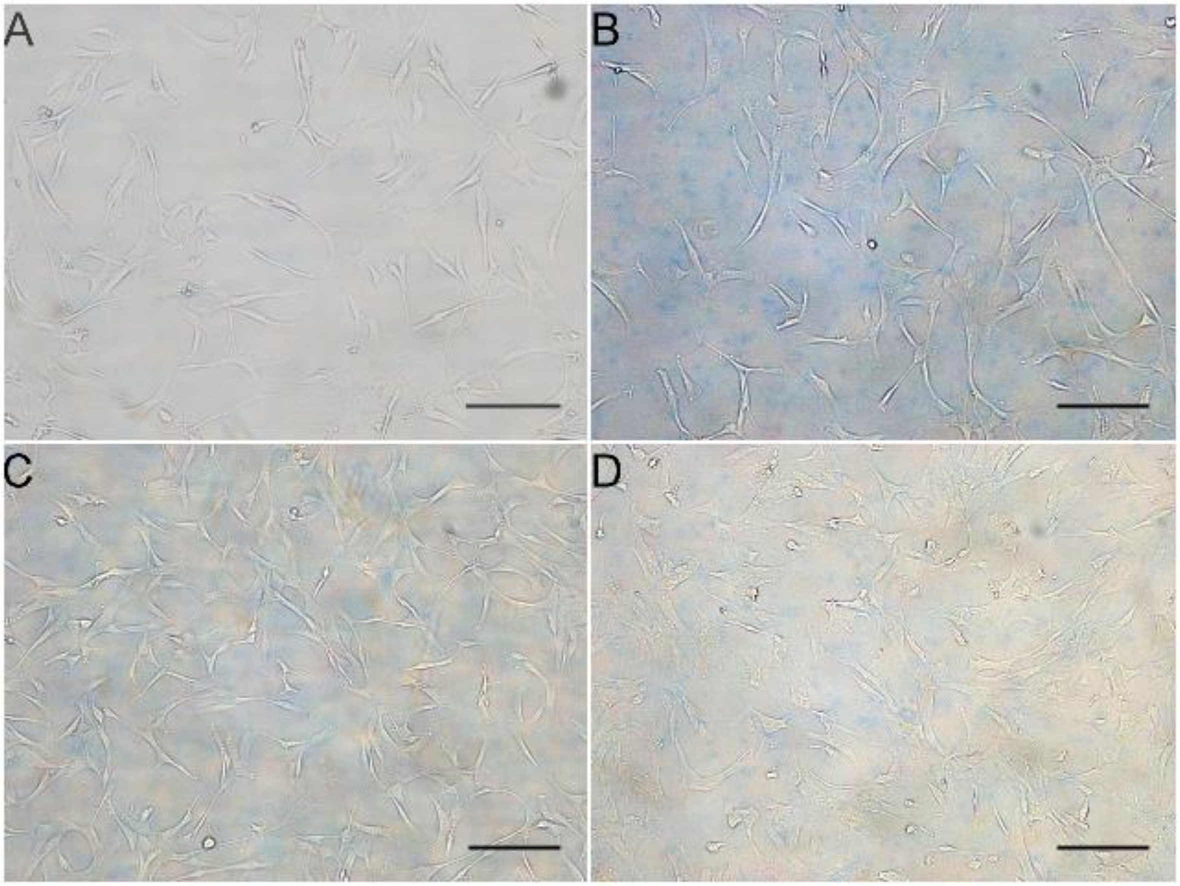

Figure S1: Fibroblastic-like morphology and adherence to plastic evidence of: A) AT-MSC, B) BM –MSC, C) SD-MSC, D) UC-MSC. Scale bar 100 μm.
