# Supplementary material for: Comparative study of the neural differentiation capacity of mesenchymal stromal cells from different tissue sources: An approach for their use in neural regeneration therapies
Source: PLoS One. 2019 Mar 11;14(3):e0213032. doi: 10.1371/journal.pone.0213032 (PMC6437714; doi:10.1371/journal.pone.0213032)
Supplement: S2 Fig — Histograms showing antigen expression in freshly (%): (A) AT-MSC, (B) BM-MSC, (C) SD-MSC and (D) UC-MSC. From left to right CD19, CD44, CD45, CD90, HLA-DR, CD29, CD73 CD105, CD73, CD34, CD105, CD11b. Black filled histogram: antigen expression; solid red line: auto-fluorescence control. (PDF) [file pone.0213032.s004.pdf]

Supporting information files

Figure S1: Immunophenotype of MSCs from different tissue source.

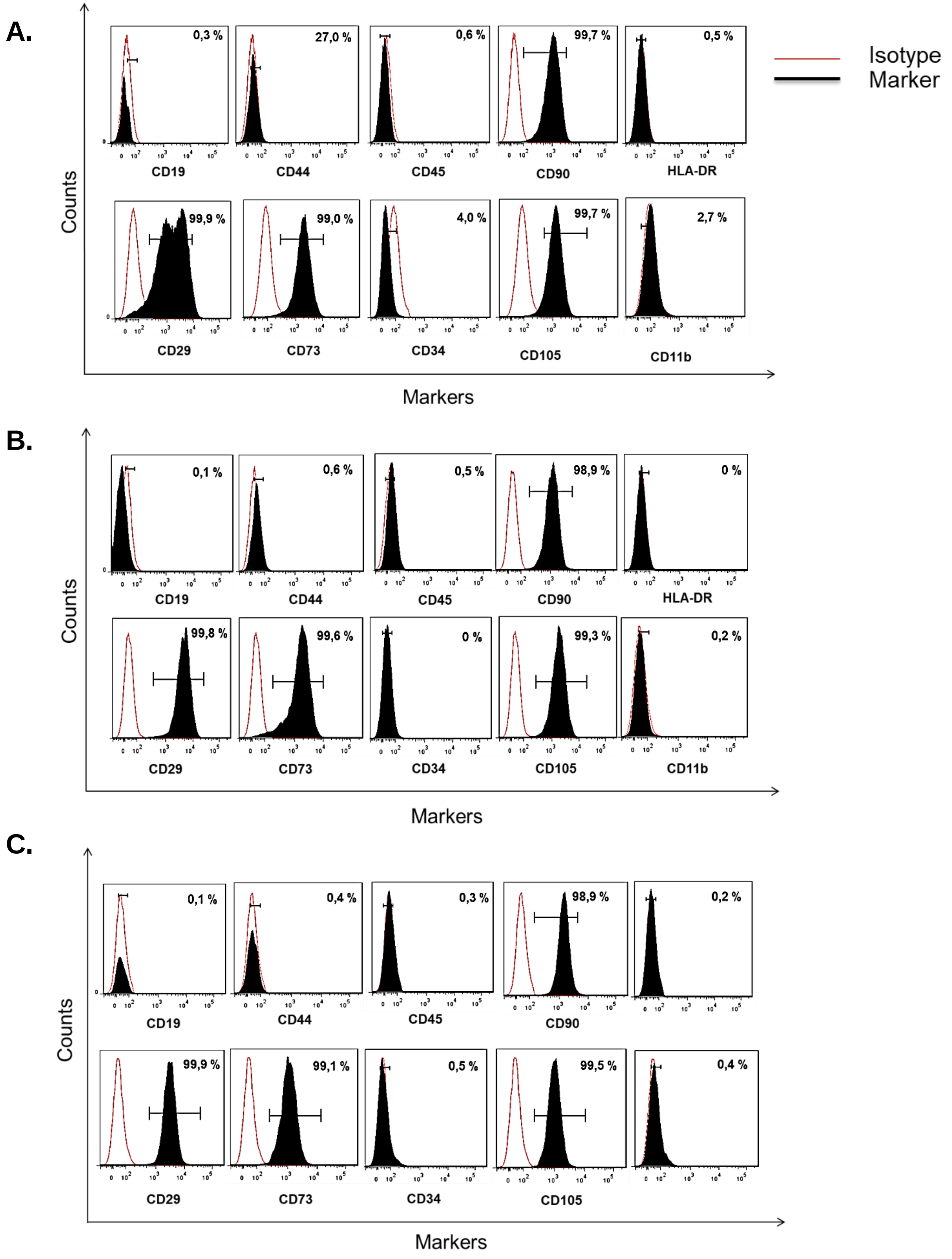

D.

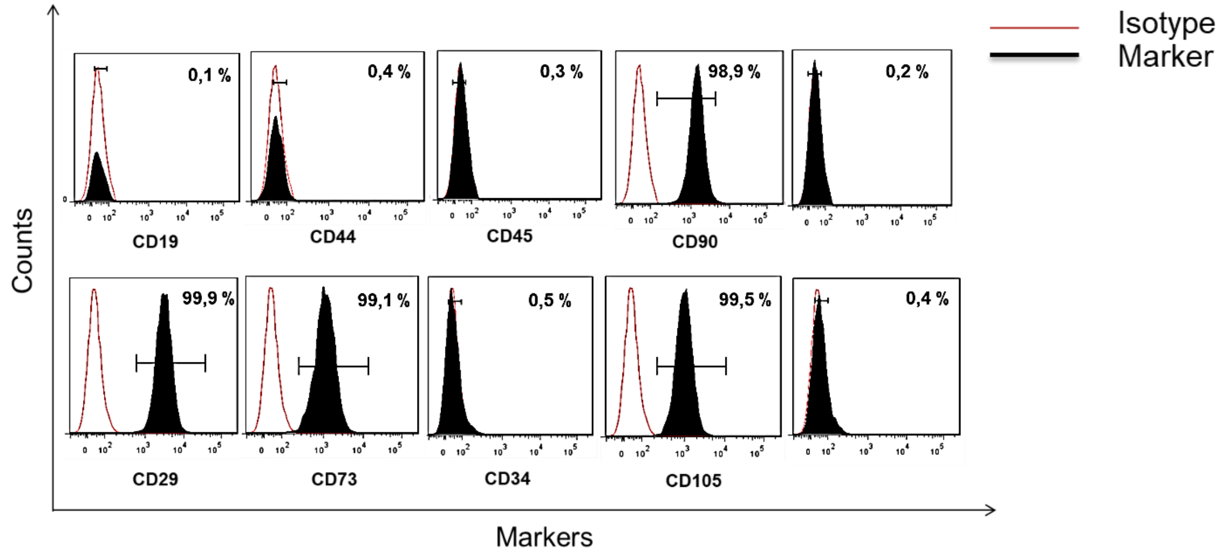

Figure S1. MSCs immunophenotype. Histograms showing antigen expression in freshly (%): (A) AT-MSC, (B) BM-MSC, (C) SD-MSC and (D) UC-MSC. From left to right CD19, CD44, CD45, CD90, HLA-DR, CD29, CD73 CD105, CD73, CD34, CD105, CD11b. Black filled histogram: antigen expression; solid red line: auto-fluorescence control
