# Supplementary material for: Comparative study of the neural differentiation capacity of mesenchymal stromal cells from different tissue sources: An approach for their use in neural regeneration therapies
Source: PLoS One. 2019 Mar 11;14(3):e0213032. doi: 10.1371/journal.pone.0213032 (PMC6437714; doi:10.1371/journal.pone.0213032)
Supplement: S5 Fig — Adipogenic differentiation of MSCs from A,B) AT-MSC, B,C) BM–MSC, C,D) SD-MSC, D,E) UC-MSC. Negative controls (B,C,D,E). All were stained with Alizarin Red. Scale bar 100 μm. (PDF) [file pone.0213032.s007.pdf]

Supporting information files

Figure S5: Osteogenic differentiation of MSCs from different tissue sources

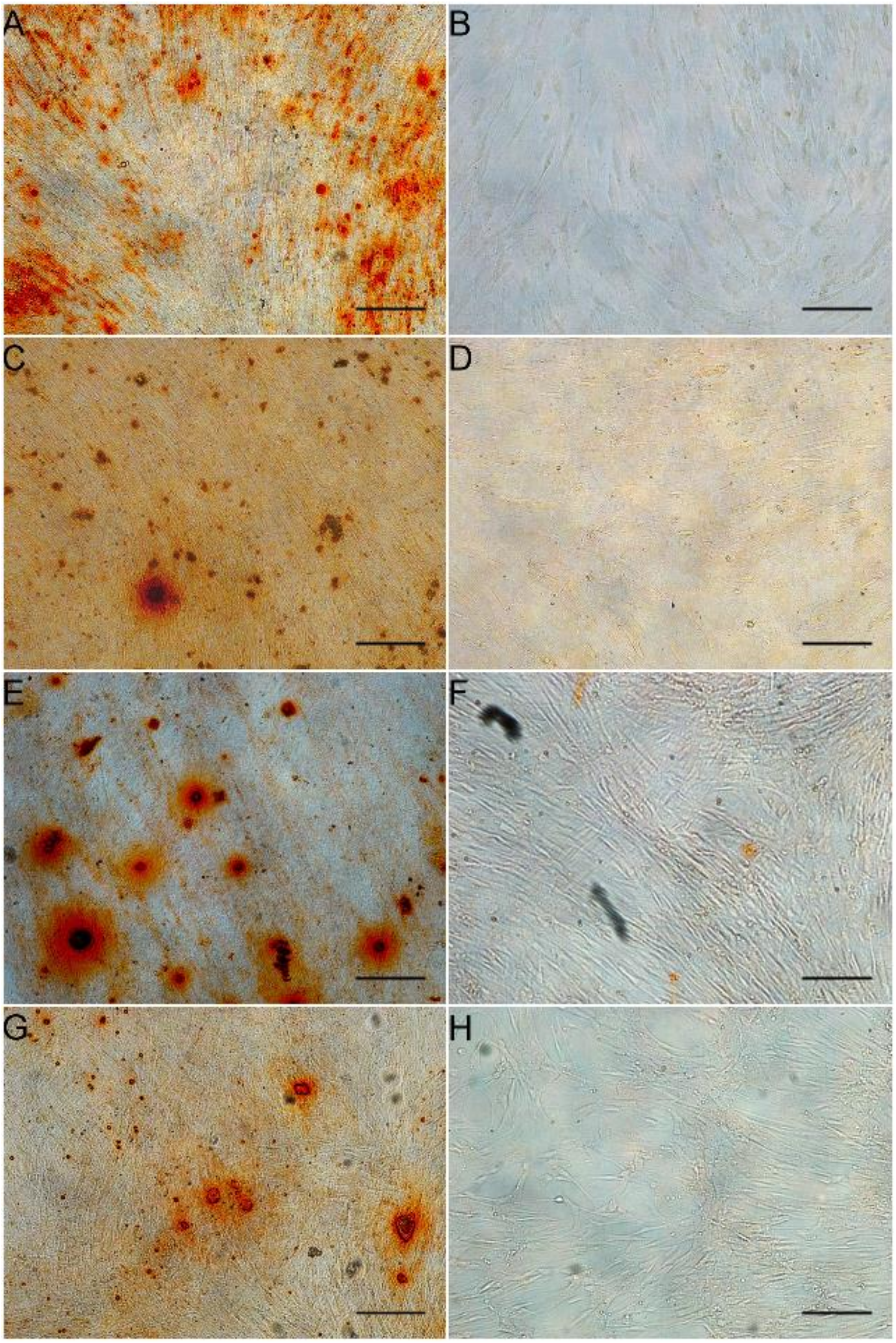

Figure S4: Osteogenic differentiation of MSCs from A,B) AT-MSC, B,C) BM –MSC, C,D) SD-MSC, D,E) UC-MSC. Negative controls (B,C,D,E). All were stained with Alizarin red. Scale bar 100 μm.
